# Examining the Effects of COVID-19 on Suicide Attempts in Budapest: A Focus on Violent and Non-Violent Attempts

**DOI:** 10.1192/j.eurpsy.2024.303

**Published:** 2024-08-27

**Authors:** M. Bérdi, N. Hajduska-Dér, B. Sebők, N. Szeifert, L. Bálint, S. Szilágyi

**Affiliations:** ^1^Psychiatry and Crisis Intervention, Peterfy Sandor Utcai Hospital-Clinic and Trauma Centre; ^2^School of PhD Studies Workgroup for Science Management, Semmelweis University; ^3^National Institute of Sports Medicine; ^4^Clinical Psychology and Addictology, Eötvös Lóránd University; ^5^Demographic Research Institute of the Hungarian Central Statistical Office, Budapest; ^6^Department of Sociology, University of Pécs, Pécs, Hungary

## Abstract

**Introduction:**

In Hungary, in contrast to most other countries, suicide deaths increased significantly during the first year of the COVID-19 epidemic (March to December 2020). Globally, the burden of emergency care in the healthcare system tended to decrease during the first period of the epidemic.

**Objectives:**

Our research aimed to evaluate the changes in the number of intentional suicide attempts by violent and non-violent means during the first two years of the epidemic, compared to the trend before March 2020 in the Budapest metropolitan area and Pest County.

**Methods:**

We analyzed psychiatric assessment reports of self-poisoning patients admitted to Péterfy Hospital’s Emergency Department and Clinical Toxicology from Jan 2019 to Dec 2021 to estimate non-violent suicide attempt trends. We analyzed patient data for violent suicide attempts treated at Dr. Manninger Jenő Trauma Centre from 2016-2021, focusing on trends during the first two years of the pandemic. Negative binomial regression estimates were used for interrupted time series analysis with Prais-Winsten regression, controlling for time and seasonal and autoregressive effects. We used change-point detection to examine the leveling of trends. The Institutional Review Board approved the research in both institutions. Approval numbers: 08-2022 (Péterfy Hospitaly) and 19-2021 (Traumatology Center).

**Results:**

The number of male non-violent suicide attempts decreased by 16.6% compared with the pre-epidemic period (p<0.001). A similar and significant decrease was observed in females and in the total population (Image 1). The female and total population trends, i.e., the decrease, were reversed by August 2020, and the male trends were reversed by October 2020. The total number of patients treated for violent suicide attempts increased significantly (p<0.05) during the first two years of the pandemic (Image 2). There was a slight increase in violent attempts in men and a small decrease in women, but these changes are not statistically significant.

**Image:**

**Figure:**
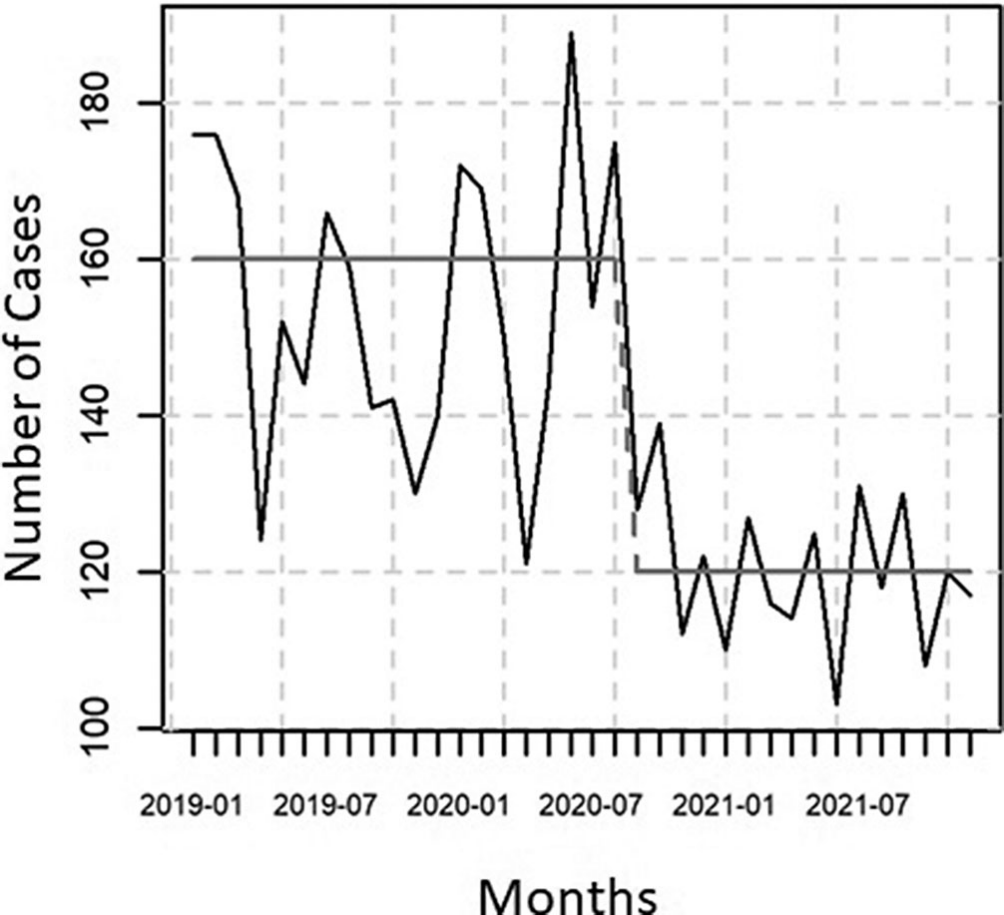

**Image 2:**

**Figure:**
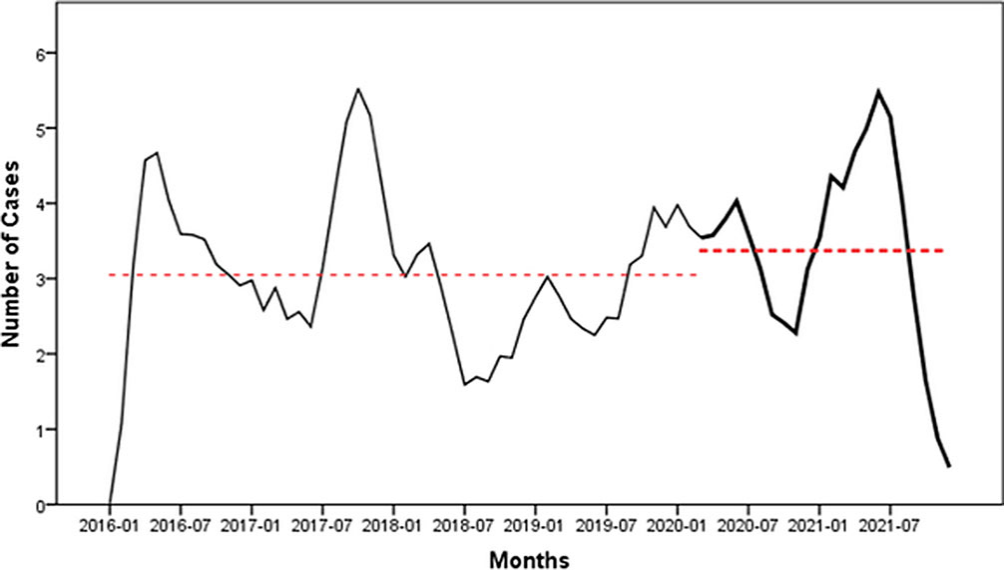

**Conclusions:**

We hypothesize that those who tried to end their life through non-violent drug use were less inclined to seek assistance because they were concerned about being hospitalized during the COVID-19 outbreak. The surge in violent attempts is striking, as it correlates with the rise in suicide fatalities documented in Hungary during the initial year of the outbreak. Our data was obtained from two prominent public hospitals in Budapest, enabling us to conduct a more concentrated and thorough examination of the circumstances in the capital.

**Disclosure of Interest:**

None Declared

